# Oral Processing of Three Guenon Species in Taï National Park, Côte d’Ivoire

**DOI:** 10.3390/biology11121850

**Published:** 2022-12-19

**Authors:** Erin E. Kane, Taylor Polvadore, Ferdinand Ouro Bele, Eloi Anderson Bitty, Ernest Kamy, Frederic Gnepa Mehon, David J. Daegling, William Scott McGraw

**Affiliations:** 1 Department of Computational Biomedicine, Boston University, Boston, MA 02118, USA; 2 Department of Anthropology, University of Arkansas, Fayetteville, AR 72701, USA; 3 The Taï Monkey Project, Taï P.O. Box 20, Côte d’Ivoire; 4 Department of Anthropology, University of Florida, Gainesville, FL 32611, USA; 5 Department of Anthropology, The Ohio State University, Columbus, OH 43210, USA

**Keywords:** *Cercopithecus*, diet, feeding ecology, guenons, ingestive behavior, oral processing, polyspecific association

## Abstract

**Simple Summary:**

Diana monkeys, Campbell’s monkeys, and Lesser spot-nosed guenons in Taï National Park, Côte d’Ivoire, are three closely related species that regularly form associations, and consume a diet with significant overlap. We took advantage of this dietary overlap and anatomical similarity to examine how closely related species process the same food items. We quantified the frequency of oral-processing behavior (use of incisors, canines, and post-canine chewing) each time foods were introduced to the mouth of these three taxa. We determined that these species use different oral-processing profiles while eating the same foods, which is surprising and intriguing since they are closely related and morphologically very similar. While our sample does not encompass the breadth of these species’ diets, it does suggest that substantial differences in the ways different taxa process food items may not be reflected in dental or facial anatomy.

**Abstract:**

Three guenon species in Taï National Park frequently form and maintain stable polyspecific associations despite significant feeding competition. This dietary overlap provides an opportunity to examine how closely related and anatomically similar taxa process the same foods. Our research examines whether the oral-processing behaviors of these guenons differ when they consume the same foods. Methods: Data on oral-processing behavior were collected on one habituated group each of *Cercopithecus campbelli*, *C. diana,* and *C. petaurista* in Taï National Park, Côte d’Ivoire from January 2016 to December 2018. We recorded the frequency with which foods were introduced to the mouth (ingestive action) and the frequency with which foods were processed using incisors, canines, and postcanine teeth. Oral-processing profiles for species-specific plant foods, fungi, and invertebrates were compared using Monte Carlo resampling. We quantified oral-processing behavior during a total of 2316 five-minute focal periods. Diana monkeys use their incisors significantly more per ingestive action than Campbell’s monkeys or Lesser spot-nosed guenons. Lesser spot-nosed guenons use their incisors more than Campbell’s monkeys. Diana monkeys also use significantly more post-canine chews per ingestive action than Campbell’s monkeys and Lesser spot-nosed guenons. Lesser spot-nosed guenons generally use fewer post-canine chews than Diana monkeys but more than Campbell’s monkeys. Canine use during feeding was rare in all three taxa. The three study species use different oral-processing profiles when consuming the same foods. These results are intriguing given the overall similarity in dental and cranial anatomy in these taxa. The oral-processing profiles we report do not encompass the full dietary breadth of all species; however, the behavioral diversity demonstrated during consumption of the same foods suggests that insight into feeding behavior is more likely obtained by examining oral processing of individual foods rather than broad food categories. Furthermore, these results underscore that important variation in feeding behavior is not necessarily associated with morphological differences in dental or craniofacial anatomy.

## 1. Introduction

**Figure 1:**
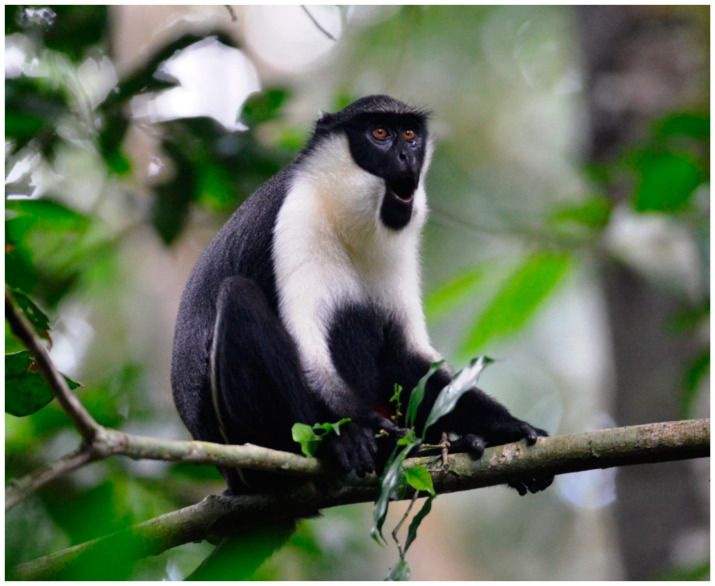
A female Diana monkey (*Cercopithecus diana*) in Taï National Park, Côte d’Ivoire. Photo by WSM.

**Figure 2:**
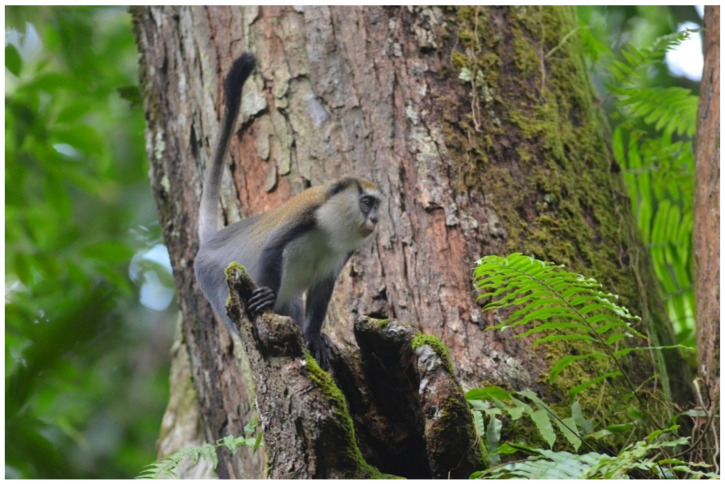
A Campbell’s monkey (*Cercopithecus campbelli*) in Taï National Park, Côte d’Ivoire. Photo by WSM.

**Figure 3:**
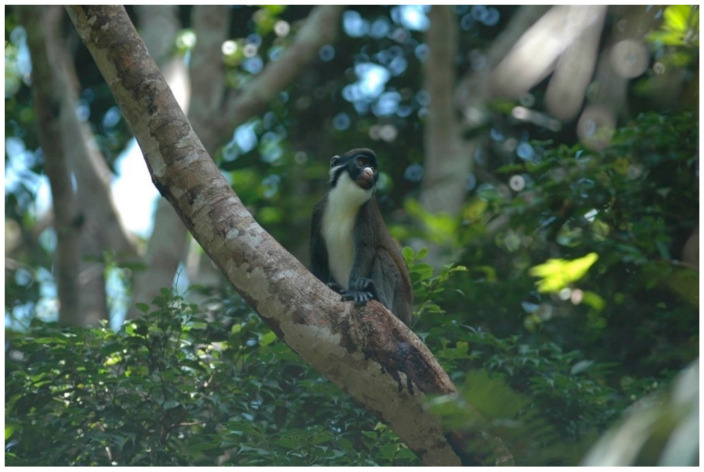
A Lesser spot-nosed monkey (*Cercopithecus petaurista*) in Taï National Park, Côte d’Ivoire. Photo by WSM.

Three guenon species ranging within Côte d’Ivoire’s Taï Forest, Diana monkeys (*Cercopithecus diana*, Figure 1), Campbell’s monkeys (*C. campbelli*, Figure 2), and Lesser spot-nosed monkeys (*C. petaurista*, Figure 3), frequently form mixed-species associations, primarily as an anti-predation mechanism against chimpanzees, leopards, and crowned eagles [1,2,3,4,5,6,7,8,9].

Ecological partitioning is one mechanism facilitating the maintenance of these associations; however, the three species still actively compete over resources. Food is the most frequently contested item, with Diana monkeys—the dominant guenon at Taï—supplanting Campbell’s monkeys and Lesser spot-nosed monkeys an average of 0.25 and 0.13 times per day, respectively [10]. When co-mingled with Diana monkeys, Campbell’s monkeys and Lesser spot-nosed monkeys, who are normally low-canopy and understory specialists, are relegated to higher forest strata. One consequence of this displacement is a change in diet: Campbell’s monkeys and Lesser spot-nosed monkeys both incorporate less fruit and more invertebrates when co-mingled with Diana monkeys than when foraging alone [4,5]. The dietary cost paid by Campbell’s monkeys and Lesser spot-nosed monkeys is offset by the sentinel abilities of Diana monkeys that presumably reduces predation risk. Diana monkey diets do not change due to these associations, but they benefit from spending less time in vigilance activities, being afforded greater foraging time [5,9,11]. Of these three species, Campbell’s monkeys seem to make the greatest use of their cheek pouches: they consistently have the most distended cheek pouches, and increase their cheek pouch use when they are not associated with Diana monkeys, hypothesized to be a response to increased predation pressure [3]. Though Campbell’s monkeys and Lesser spot-nosed monkeys undergo dietary shifts as a consequence of associating with Diana monkeys, the species maintain substantial dietary overlap [2,4,5,10]. The fact that these species consume the same foods, including when co-mingled, provides an opportunity to investigate whether oral-processing behaviors vary between closely related taxa eating the same foods.

This question is particularly germane for guenons because there is extensive overlap in the range of craniodental variation within and between species, a reality which has complicated attempts to associate species-specific morphological features with diet [12,13]. Guenon species exhibit statistical differences in dental morphology; however, craniodental anatomy among guenons appears to map poorly onto ecological variables [12,13,14]. Interspecific comparisons have demonstrated that, while there may be central tendencies characterizing guenon incisor and molar morphology, the range of variation within a species makes segregating by species challenging or impossible [12,14,15]. Since these species are similarly sized (2.7–5.2 kg) [16] and closely related, there are very few distinguishing features to discriminate their crania and dentition and it is challenging to confidently ascribe *Cercopithecus* skeletal and dental remains found at Taï to particular species [17].

The few attempts to relate features of *Cercopithecus* craniodental anatomy to differences in feeding behavior have produced enigmatic and often conflicting results [14,15,18]. Though guenon diets may not vary enough for oral-processing behaviors to require functionally relevant differences in morphology, field data continue to highlight dietary variation within *Cercopithecus* [19,20,21,22]. However, the use of broad diet categories as opposed to behavioral differences has compromised our ability to identify functionally meaningful aspects of feeding anatomy. For example, the feeding ecology of many guenon species is only known at the level of the broad dietary category [19,20,21,22], limiting the strength or specificity of conclusions from morphological research including on these taxa. As more species become the subject of intensive ecological study, one way to better identify ecologically meaningful elements of craniodental morphology is to examine how different foods are processed and whether species process the same foods in similar ways [23,24,25,26].

This approach has yielded important insights into relationships between primate feeding behavior, ecology, and morphology. For example, Yamashita [27,28] demonstrated that food size, shape, and mechanical properties influence oral processing in *Lemur catta* and *Propithecus verreauxi*, showing that food size and shape, though not necessarily toughness, are important indicators of whether lemurs begin the process of ingestion with anterior or posterior teeth. At one site, *Lemur catta* rely heavily on *Tamarindus indica* fruit, which is protected by a particularly tough and hard pod [27,28,29,30,31]. Processing this fruit induces repeated loads on *L. catta’s* thin-enameled teeth, as they repeatedly bite the pod to induce cracking; this is likely a significant contributor to high rates of dental wear and tooth loss in this *L. catta* population [32]. Observations of the ingestive behaviors and mechanical properties of foods eaten by *Sapajust libidinosus* in Brazil [33,34,35] coupled with captive research on mandibular strain and muscle fiber architecture in this species [36,37] suggest that ingestive behaviors, particularly those related to high mandibular strain, are important pressures on craniodental morphology in robust capuchin monkeys. Dental and cranial traits of *Gorilla gorilla gorilla*, including thick enamel and increased molar-shearing crests, are generally assumed to facilitate the processing of tough, fracture-resistant foods such as leaves and fibrous vegetation [38]. However, recent observations of the oral-processing behaviors of *Gorilla gorilla gorilla* found surprising evidence of feeding on hard woody seeds, indicating that gorillas exhibit more dietary flexibility than previously assumed [38].

Similarly, long-term research in the Taï Forest has shown how oral processing can be used to interpret aspects of craniodental morphology [17,39,40,41,42,43,44,45,46,47,48,49,50,51,52,53,54,55] and the consequences of processing different foods, including dental macro- and microwear [17,54,55]. Pairing morphological studies with field observation of idiosyncratic or conspicuous feeding behaviors (e.g., the isometric bite of *Cercocebus atys* eating *Sacoglottis gabonensis* seeds [47,49,50] or the incisal gnawing of *Pentaclethera macrophylla* pods by *Colobus polykomos* [42,43,44,51]) provided the necessary context to interpret morphology. Both observational and experimental research underscore that oral-processing behavior, rather than characteristics of a food item per se, mediates morphology [25]. For example, both sooty mangabeys and wild chimpanzees in the Taï Forest eat the hard, tough seeds of *Coula* nuts [45,56]: chimpanzees manually process the seeds with tools before ingestion; mangabeys simply bite them.

In this study, we focus on three species that are less anatomically divergent than the subjects of previous comparative studies at Taï, which focused primarily on distinctive feeding behaviors and morphologies of the colobines and mangabeys. If these studies included *Cercopithecus* spp., they either only included Diana monkeys [47] or considered all three *Cercopithecus* as one group of frugivores due to the overall craniodental anatomical similarity between the taxa [17], and because there are not any species-specific oral-processing behaviors that obviously distinguish one taxon from another. While the guenon radiation is an example of great diversity in terms of feeding ecology [19,20,21,22], this ecological diversity is mapped onto overall morphological uniformity [12,13,14], suggesting overall behavioral uniformity in the ways that guenons process the foods they eat. With this in mind, we use oral-processing data on three Taï Forest guenons to address the following general question: do different species process the same foods in similar ways? Given the homogeneity of guenon cranial and dental anatomy, our general prediction is that the oral-processing profiles of these taxa will be similar, especially since we are restricting our examinations to foods consumed by multiple taxa. Observing differences in oral-processing behaviors, despite overall morphological homogeneity in these taxa, would further complicate an assumed form–function relationship in *Cercopithecus* craniodental morphology.

## 2. Materials and Methods

### 2.1. Study Site and Species

Data were collected in Taï National Park, Côte d’Ivoire. The park is comprised of 330,000 ha of protected forest surrounded by a matrix of agricultural plantations and villages and located in southwestern Côte d’Ivoire, approximately 25 km from the Liberian border [57,58]. The park experiences two wet seasons (April to June and September to October) and two dry seasons (November to March and July to August), receiving an average of 1893 mm of rainfall annually [59]. We collected data from three habituated groups, Diana monkeys, Campbell’s monkeys, and Lesser spot-nosed guenons, within the study area of the Taï Monkey Project [57].

During the study period, the focal Diana monkey group included 7 adult females and associated offspring; the adult male was replaced in December 2016 and January 2017 [22]. Diana groups average at 23.5 individuals including one adult male, 7–13 adult females, and associated subadults, juveniles, and infants [11]. Groups of Campbell’s monkeys (average = 9.3) and Lesser spot-nosed guenons (average = 11.3) are substantially smaller than Diana monkey groups, each with one adult male, 4–8 adult females, and associated subadults, juveniles, and infants [11]. Though the Taï guenons’ digestive anatomy has not been studied in detail, it is likely that, as in other *Cercopithecus* species, they have a relatively long gut passage time (20.6 +/− 12.8 SD hours in captive *Cercopithecus mitis*) [60].

Behavioral data were only collected on adults, who were readily distinguished from juveniles and subadults by overall body size, nipple size, and vocalizations. Campbell’s monkeys and Diana monkeys were individually identifiable by features of the pelage, tail, and nipples or external genitalia, though Diana monkeys were not individually identified in every focal due to visibility challenges; Lesser spot-nosed guenons were not able to be individually identified beyond age- and sex-class (Table 1).

Based on samples of wild-shot individuals in Sierra Leone and museum specimens, mean body weights of male and female Diana monkeys are 5.2 kg and 3.9 kg, Campbell’s monkeys are 4.5 kg and 2.7 kg, and Lesser spot-nosed guenons are 4.4 and 2.9 kg [16]. Observations of Diana monkeys included only adult females. Observations of Campbell’s monkeys and Lesser spot-nosed guenons included both adult males and adult females (Table 1). Because we found no significant difference in males’ and females’ frequency of incisions or post-canine chews per ingestive action, we pooled data from adult males and females for all analyses (Wilcoxon signed-rank tests, Campbell’s monkeys: incisions per ingestive action: W = 4308, *p* = 0.56; postcanine chews per ingestive action: W = 4167, *p* = 0.94. Lesser spot-nosed guenons: incisions per ingestive action: W = 1423, *p* = 0.41; postcanine chews per ingestive action: W = 1952, *p* = 0.33).

### 2.2. Oral-Processing Data Collection

We generated oral-processing profiles from observations made during focal follows conducted by three field assistants (FOB, EK, FMG) and the first author (EEK) who were trained by the last author (WSM). Data were collected on adult Diana monkeys from April 2016–September 2017 and a subset of these data has been reported previously [39]. Data were collected on adult Campbell’s monkeys and Lesser spot-nosed guenons from March 2017–December 2018. These data were collected during regular full-day follows, which typically occur from sunrise to sunset. Observers were between 3–30 m from the focal animal, and binoculars were used when focal individuals were more than 10 m away. To avoid short-term repeated sampling of non-independent events, individuals were never sampled more than once per hour when individual identification was possible (Diana monkeys, Campbell’s monkeys). When individual identification was not possible (Lesser spot-nosed guenons and, occasionally, Diana monkeys), individuals of the same age- and sex-class were never sampled more than once an hour.

Data were collected during five-minute focal periods. A focal period began when food was introduced to the oral cavity, either by the hand or when the monkey bit the food item directly from a substrate. Using a notebook, we recorded the food type ingested using the following categories: ripe and unripe fruit, young and mature leaves, flowers, fungi, invertebrates, stems that had been stripped of leaves, and other material. Where possible, we further identified food items to species level, based on long-term research on guenon diets in the Taï Forest [10,22].

During each focal period, we recorded the number of ingestive actions, defined as the number of times food was introduced to the oral cavity [40]. We did not count the number of food items introduced to the mouth per ingestive action, but if multiple kinds of foods were eaten together (for example, leaves and fruit ingested in the same bite) that focal was excluded from further analysis. We recorded incisions each time an individual used their anterior teeth to bite pieces from a food item, or bite food items into smaller pieces. Canine use was recorded when individuals punctured food items with their canines, an activity distinguishable from incision because of the increased gape required. We recorded post-canine chews as the number of chewing cycles involving premolars and molars.

*Cercopithecus* monkeys have amylase-secreting cheek pouches in which they cache food [3,61]. Because we could not reliably record the frequency with which foods were cached or removed from cheek pouches during focals, and because foods may not have been fully chewed before the next ingestive action, it is possible that we underestimated the frequency of oral-processing behaviors per ingestive action. Conversely, we may have overestimated oral-processing behaviors if a pouched mechanically challenging food was mixed with a newly ingested soft food.

### 2.3. Analytical Methods

We divided the frequency of tooth use (incisors, canines, and postcanine teeth) by the number of ingestive actions for each focal. This provided some standardization to compare across focals, since introducing more food items to the mouth requires more processing than introducing a single food item. We reported oral-processing profiles as mean tooth use frequencies per ingestive action for our five food categories and species-specific plant parts. There was a marked disparity in sample size between species (Table 2).

To account for this imbalance, we used a Monte Carlo resampling procedure to compare oral-processing profiles for all pair-wise species contrasts. For food categories and species-specific food items, we pooled incisions and post-canine chews across focal periods per guenon species. Because sample sizes were unequal for all contrasts, we employed a conservative procedure to explore whether small N in a given taxon could be explained as an outcome of resampling at smaller N from the better represented taxon. In other words, the taxon with the larger N was resampled to the smaller N without replacement in over 1000 trials. *p*-values reflect the number of trials in which the resampled mean differed by the same sign (positive or negative) from the empirical mean observed in the taxon with the smaller N; we report the direction of the significant difference. Tests were one-tailed; we tested whether the magnitude of the observed difference (accounting for the sign of the difference) was met or exceeded by resampling the taxon with the higher N to the N of the contrasted taxon in over 1000 trials. Thus, *p* = 0.5 indicates that the magnitude of the observed difference met or exceeded the resampled difference in half the trials. *p* = 0, by contrast, indicates that the resampled statistic never met or exceeded the observed mean difference (this is equivalent to *p* < 0.001 reported in conventional statistics). Although we report on mean canine use, because this behavior was extremely rare we did not compare it statistically. Data were analyzed in R using the base package [62]; figures were made using ggplot2 [63]. For within-species comparisons (i.e., males versus females discussed in Section 2.1), we utilized the non-parametric Wilcoxon test in R using the base package [62].

### 2.4. Data Availability and Ethical Statement

These data are available from the corresponding author upon request. Data collected for this study were observational and followed the American Society of Primatologists’ principles for the ethical treatment of nonhuman primates as well as the guidelines of permit-granting bodies in Côte d’Ivoire and the IACUC at The Ohio State University, protocol number 2008A0051-R4.

## 3. Results

**Figure 4:**
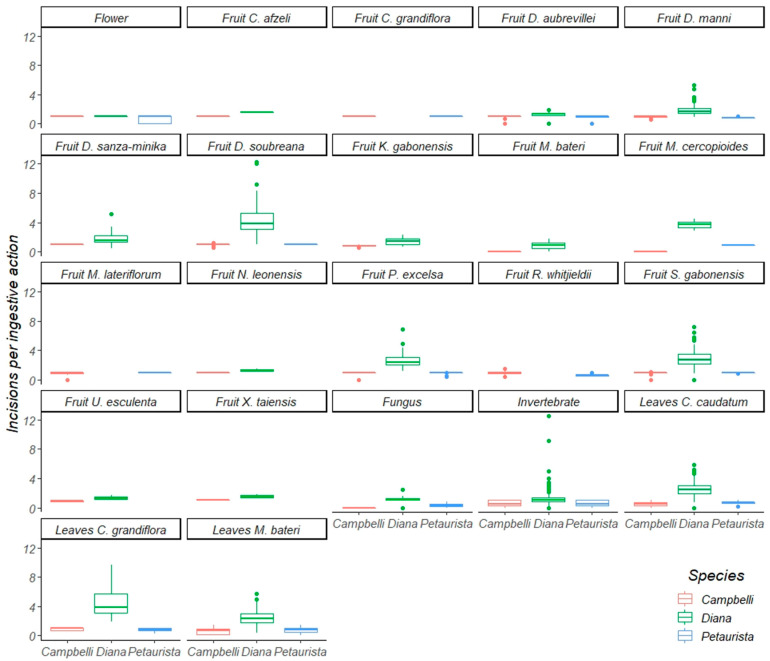
A boxplot of incisions per ingestive action of Diana monkeys, Campbell’s monkeys, and Lesser spot-nosed monkeys while eating the same food items during this study. The line inside each box represents the median; whiskers represent the 25 and 75 percentiles.

**Figure 5:**
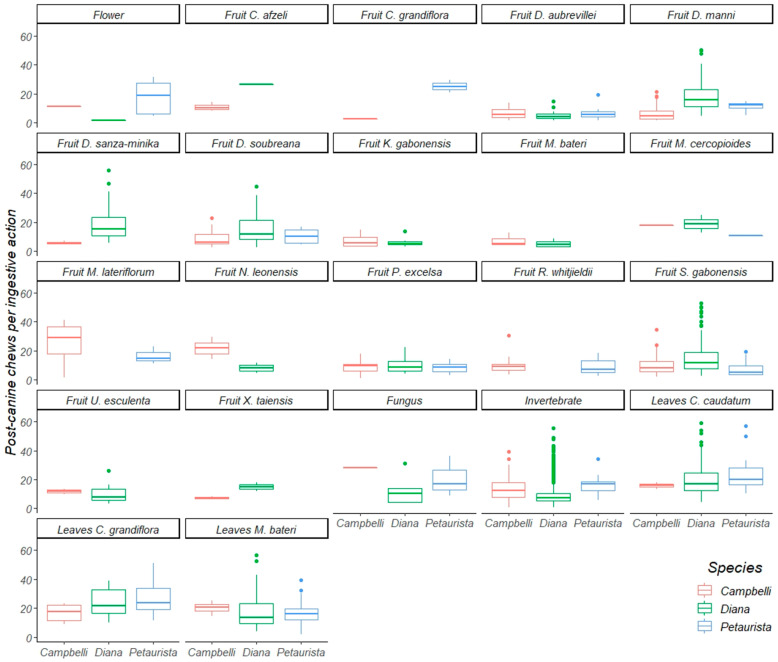
A boxplot of postcanine chews per ingestive action of Diana monkeys, Campbell’s monkeys, and Lesser spot-nosed monkeys while eating the same food items during this study. The line inside each box represents the median; whiskers represent the 25 and 75 percentiles.

Between April 2016 and December 2018, we quantified oral-processing activity across the three guenon taxa during a total of 2316 focal periods. This includes 288 focal periods of Campbell’s monkeys, 1838 focal periods of Diana monkeys, and 182 focal periods of Lesser spot-nosed guenons (Table 2). During this period, we collected focal observations of Campbell’s monkeys eating sixteen fruit species, two young and three mature leaf species, invertebrates, fungi, and seeds. Diana monkeys were observed eating 14 fruit species, four young leaf species, two mature leaf species, invertebrates, fungi, flowers, and leaf petioles. Lesser spot-nosed guenons were observed eating nine fruit species, four mature leaf species, two young leaf species, invertebrates, flowers, fungi, and one seed species. We emphasize that the total dietary breadth of these species is greater than that reported here [10,23], and that this study is restricted to those foods consumed by two or more species (Table 3), which represents a subset of the larger dataset (Supplemental Table S1). We were able to statistically compare consumption of 13 fruit species, 5 leaf species, invertebrates, fungi, and flowers (Table 4) in terms of their incisions (Figure 4) and post-canine chews (Figure 5) per ingestive action. We did not statistically examine the role of inter-individual variability due to constraints of sample size (Supplemental Figures S1 and S2).

### 3.1. Oral Processing of Fruit

Diana monkeys and Campbell’s monkeys ate 14 fruit species in common (Table 3 and Table 4). Diana monkeys processed eleven of these with significantly more incisions per ingestive action than Campbell’s monkeys, and two with (statistically) the same frequency of incisions per ingestive action. Diana monkeys processed ten fruit species with significantly more postcanine chews per ingestive action than Campbell’s monkeys, and four species with the same frequency of postcanine chews per ingestive action. Diana monkeys and Lesser spot-nosed guenons ate six fruit species in common (Table 3 and Table 4) and, in every case, Diana monkeys used significantly more incisions per ingestive action than Lesser spot-nosed guenons. Diana monkeys also used significantly more postcanine chews per ingestive action than Lesser spot-nosed guenons while processing five fruits eaten in common; only one species was processed with the same frequency of postcanine chews. Campbell’s monkeys and Lesser spot-nosed guenons ate nine fruit species in common (Table 3 and Table 4). Lesser spot-nosed guenons processed five fruit species with significantly more incisions and more postcanine chews per ingestive action than Campbell’s monkeys, and four species with the same frequency of incisions and postcanine chews per ingestive action.

### 3.2. Oral Processing of Foliage

Diana monkeys and Campbell’s monkeys ate four species of young and mature leaves in common (Table 3 and Table 4). Diana monkeys processed all four species using significantly more incisions per ingestive action than Campbell’s monkeys, and two of the four species were processed with significantly more postcanine chews per ingestive action. These two guenons used the same frequency of postcanine chews per ingestive action during the consumption of two leaf species. Diana monkeys and Lesser spot-nosed guenons ate five of the same species of young and mature leaves and leaf petioles (Table 3 and Table 4). Diana monkeys used more incisions and postcanine chews per ingestive action than Lesser spot-nosed guenons when processing four leaf species, and both species used the same incision and postcanine chew frequency per ingestive action when processing the fifth. Campbell’s monkeys and Lesser spot-nosed guenons ate four leaf species in common (Table 3 and Table 4). There was no interspecific difference in incisions per ingestive action while processing leaves, but when processing three of the four species, Lesser spot-nosed guenons used more postcanine chews per ingestive action than Campbell’s monkeys. Postcanine chewing frequency per ingestive action was the same for the fourth leaf species.

### 3.3. Oral Processing of Invertebrates, Fungi, and Flowers

Diana monkeys and Campbell’s monkeys both ate invertebrates and flowers (Table 3 and Table 4). Diana monkeys used more incisions per ingestive action than Campbell’s monkeys when processing both food types. Diana monkeys also used more postcanine chews per ingestive action than Campbell’s monkeys while processing flowers; both species used the same frequency of postcanine chews to process invertebrates. Diana monkeys and Lesser spot-nosed monkeys ate invertebrates, fungi, and flowers (Table 3 and Table 4). When processing all three foods, Diana monkeys used more incisions per ingestive action than Lesser spot-nosed guenons, but used the same frequency of postcanine chews per ingestive action. Lesser spot-nosed guenons and Campbell’s monkeys ate invertebrates and flowers (Table 3). When processing both invertebrates and flowers, Campbell’s monkeys and Lesser spot-nosed guenons used the same frequency of incisions per ingestive action, but Lesser spot-nosed guenons used more postcanine chews.

## 4. Discussion

In this study, we examined the oral-processing behavior of three closely related, sympatric guenon species when they processed the same food items. Compared to their congeners, Diana monkeys use more incisions per ingestive action when consuming the same leaves, invertebrates, and flowers, and more post-canine chewing per ingestive action when eating most fruits. Secondly, Campbell’s monkeys and Lesser spot-nosed guenons process food items in a more similar way to each other than Diana monkeys. When differences do occur, Lesser spot-nosed guenons use more incisions and chews per ingestive action than Campbell’s monkeys for half of the fruits and approximately ¾ of the leaves that both species eat, as well as more post-canine chewing while eating leaves and flowers. Campbell’s monkeys very rarely engaged in more oral processing per ingestive action than either of the other species. Thus, when there were interspecific differences in oral processing of the same food item, Diana monkeys engaged in the most incision and post-canine chewing and Campbell’s monkeys the least, with Lesser spot-nosed guenons being intermediate. Our findings thus indicate that *even when ingesting the same foods*, these three species’ oral-processing profiles differ. Such differences are apparent despite restricting comparisons to foods eaten in common; we would likely detect greater differences in oral-processing profiles across the full range of these species’ diet [4,10].

We caution that our sample of Campbell’s monkeys’ and Lesser spot-nosed guenons’ oral-processing behavior is limited in both sample size and dietary scope. For example, we are likely missing foods eaten only by Campbell’s monkeys and Lesser spot-nosed monkeys, especially those eaten when their diet shifts in response to competitive exclusion during association [4]. These food items may be less preferred, perhaps because they are more mechanically protected or offer different challenges when eating. For example, *Rothmania whitjeldii,* a fruit eaten by Lesser spot-nosed guenons and Campbell’s monkeys (but not Diana monkeys), has a tough outer skin and hard pulp protecting the soft inner flesh eaten by these monkeys and poses a significant mechanical challenge to open it (TMP, unpublished data). Observing how Lesser spot-nosed guenons process other leaves that they regularly eat would likely increase the overall number of chewing cycles in *C. petaurista* (assuming folivory reliably tracks chewing frequency). Expanding our sample of oral-processing behavior across the full breadth of the Campbell monkey and Lesser spot-nosed guenon diets at Taï may therefore highlight further differences or identify unrecognized areas of congruence between taxa in their overall oral-processing profiles. Diana monkeys, who specialize in ripe fruit rather than hard seeds [22], exhibit a similar degree of symphyseal and postcanine bone remodeling as hard-object-specializing sooty mangabeys [47]. Perhaps a broader comparative sample would confirm this, despite their diet incorporating fewer leaves than the sympatric Lesser spot-nosed monkey’s [4]. A broader comparative sample may also demonstrate that, when eating foods not shared with Diana monkeys, Lesser spot-nosed guenons and Campbell’s monkeys engage in more post-canine chewing. Alternately, a broader sample may identify previously unsuspected mechanical challenges in Diana monkeys’ diets. Fitting Campbell’s monkeys and Lesser spot-nosed guenons into this complex picture will provide additional insights into the relationship between ingestive behavior, diet, and morphology. In addition to expanding our comparative sample, future work will aim to collect food mechanical properties for a wide array of food items, including those shared between taxa to address the role of food mechanical properties in oral processing [22]. Future work will also expand our sample to include juvenile and subadults of all species in our sample, highlighting the role of ontogeny, allometry, and ecological competence in oral-processing behavior among these taxa.

The current study provides important context to understand the relationship between primate feeding ecology, ingestive behavior, and morphology. Given the uniformity of the *Cercopithecus* mandibular, maxillary, and dental anatomy [12,13,14], it is surprising—under the premise that morphology tracks performance directly—that we found consistent, significant differences in the oral-processing regimes of three guenons when consuming the same foods. Previous research has struggled to associate elements of guenon diet with craniodental anatomy [12,13,14,15,18]. One reason for this is the tendency to use feeding categories (e.g., % of fruit vs. leaves consumed) to frame hypotheses about jaw and tooth morphology in this genus, especially in the absence of more detailed data about dietary composition, food mechanical properties, and ingestive behavior [19,20,21,22]. Given the potential for dramatic variation in the mechanical properties and associated loading regimes while processing foods in the category “fruit” or “leaf”, it is unsurprising that mapping feeding ecology to cranial morphology has been unsatisfactory at best [18,19,20,21,22]. Based on assumed relationships between food mechanical properties of diet categories, oral processing, and loading regimes, we would have expected frugivorous *C. diana* to engage in less oral processing than the relatively more folivorous *C. petaurista* and *C. campbelli*, with concomitant morphological signatures. Given the homogeneity of guenon cranial and dental morphology, we predicted that these three taxa would process the same foods in the same way. The oral-processing profiles generated during the present study demonstrate that hypotheses about feeding morphology in these three guenon species based on hypothetical loading regimes related to broad dietary categories are incorrect. In fact, we found that Diana monkeys consistently engage in more oral processing per ingestive action, engaging their incisors and their post-canine teeth at a greater frequency with each ingestion than Lesser spot-nosed guenons or Campbell’s monkeys.

That oral-processing behavior does not bear out assumptions based on diet and morphology is not necessarily surprising as, as experimental research [37] has demonstrated, differences in mandibular strain patterns (as a proxy for bite force [64]) cannot be reliably linked to differences in food mechanical properties, and oral-processing behavior (such as chewing side, or the number of chewing cycles), has a stronger effect on mandibular strain [37]. In these analyses, we assumed that the number of incisal bites and cyclical loads during chewing are reasonable proxies for effort [37,64]). While there is good evidence suggesting that initial incisal bites may entail more work than masticatory cycles [37], this is a simplifying assumption, given our inability to adequately determine loads during oral processing.

Guenon craniodental morphology may be general enough that the demands of each species’ oral-processing profile do not leave distinctive morphological traces. It is possible that hitherto unrecognized differences in dental anatomy can explain different rates of incision and/or post-canine chewing. Indeed, one study highlights a potential mismatch between what one of our study’s species consumes and how its teeth wear. Bunn and Ungar [65] found that *C. campbelli* exhibited tooth wear patterns (high occlusal angularity) similar to those in two highly folivorous Taï colobines, *Colobus polykomos and Piliocolobus badius*, though leaves contribute only a small amount to their diet. This “mismatch” may vanish when considering that insects comprise a large portion of the *C. campbelli* diet. Chitin may present similar mechanical challenges, as with leaves in certain contexts [66,67]. These mismatches may only be apparent because we are assuming that morphological variation is precisely mapping onto the mechanical demands of feeding. Liem’s paradox [68] encourages us to be aware of biases in terms of how we expect morphological differences to mesh neatly with dietary variation. The paradox emerges because we expect that highly derived morphology reflects derived behavior (in this case, dietary specialization). Ungar has provided examples of the paradox from living and fossil primates in terms of dental morphology [69,70].

Considering ingestive behavioral variation in wild primate populations rather than simply quantifying diets or working only in a laboratory context has provided important insights to the study of primate craniodental morphology, pointing towards selective pressures on morphology [27,28,29,30,31,32,33,34,35,40,45], highlighting surprising dietary challenges [38], and examining how oral-processing behavior relates to community ecology [23,32,51]. Because our data do not characterize oral-processing profiles across the dietary regimes of Diana monkeys, Campbell’s monkeys, and Lesser spot-nosed guenons, we are reticent to use these data to directly compare guenon oral-processing profiles to other primate taxa. However, by demonstrating that three anatomically similar, closely related taxa process the same foods in distinct ways, our results add to the growing body of work demonstrating the complexity of relating oral-processing behavior to feeding ecology and craniodental morphology.

## 5. Conclusions

We have found that, contrary to predictions, three closely related species with similar craniodental anatomy process the same foods in significantly different ways. We are skeptical that a more granular investigation of craniofacial anatomy will uncover cryptic variation in biomechanical performance variables. That the facial skeleton of these monkeys is up to the task of processing their respective diets is unassailable. On the other hand, more research is needed to characterize these species’ diets in terms of mechanical properties, oral-processing behaviors, and digestive anatomy across the full breadth of their diet and ontogeny. Comparative morphological approaches often assume that natural selection is targeting one or a finite set of ecological variables and that this target is the same across taxa. The results reported here suggest that this assumption does not clarify the relationship between feeding behavior, diet, and ingestive performance variables among the sampled guenon species.

## Figures and Tables

**Table 1:** The number of focals during this study for each species and, when possible, each individual. All observations of individuals who could not be identified beyond age- and sex-class were pooled by species; focals of “unknown” animals likely include multiple individuals.

Species	Age–Sex Class	Individual ID	Number of Focals
*C. diana*	Adult female	Unknown	300
*C. diana*	Adult female	Agnes	207
*C. diana*	Adult female	Cheri	203
*C. diana*	Adult female	Emi	165
*C. diana*	Adult female	Eva	321
*C. diana*	Adult female	Nina	201
*C. diana*	Adult female	Sonia	202
*C. diana*	Adult female	Veronique	239
*C. campbelli*	Adult female	Agathe	75
*C. campbelli*	Adult female	Caro	11
*C. campbelli*	Adult female	Florence	126
*C. campbelli*	Adult female	Lucie	73
*C. campbelli*	Adult female	Ode	70
*C. campbelli*	Adult male	Domi	40
*C. petaurista*	Adult female	Unknown	198
*C. petaurista*	Adult male	Unknown	118

**Table 2:** Focal observations of oral-processing behavior for three guenon species, by food.

Species	Focals	Flowers	Fruit	Invertebrates	Leaves	Fungi	Seeds	Stems
*C. diana*	1838	1	435	1161	227	8	0	6
*C. campbelli*	395	1	239	104	21	1	14	14
*C. petaurista*	316	7	67	24	192	3	3	20

**Table 3:** We report the number of focals by food type and by species, the number of ingestive actions, and median 1. incisor use, 2. canine use, and 3. post-canine chews per ingestive action for foods eaten by at least two guenon species.

	Diana Monkeys (*C. diana*)					Campbell’s Monkeys (*C. campbelli*)					Lesser Spot-Nosed Monkeys (*C. petaurista*)				
	Focals (N)	Action (N)	I/A ^1^	C/A ^2^	PC/A ^3^	Focals (N)	Action (N)	I/A	C/A	PC/A	Focals (N)	Action (N)	I/A	C/A	PC/A
**All focals**	1838	21,275	1.27	0.00	8.82	395	4468	1.00	0.00	9.58	315	3099	0.82	0.00	14.39
**Fruit**	582	11,657	2.07	0.00	19.48	223	2574	0.86	0.01	10.15	73	1156	0.88	0.05	9.91
*Cersalia afzeli*	1	8	1.63	0.00	26.38	4	13	0.88	0.00	10.43					
*Coelecarion oxycarpum*	1	10	1.10	0.00	4.30	1	12	1.00	0.00	5.50					
*Culcasia grandiflora*						1	21	1.00	0.00	2.80	2	11	1.00	0.00	25.13
*Dialium aubrevillei*	27	789	1.30	0.00	8.90	15	156	0.91	0.00	6.96	7	92	0.85	0.00	7.21
*Diospyros manni*	148	2547	1.92	0.00	37.74	25	241	0.95	0.02	7.05	7	78	0.85	0.15	11.32
*Diospyros sanza-minika*	22	305	1.90	0.00	21.14	3	33	1.00	0.00	5.75					
*Diospyros soubreana*	61	936	4.58	0.00	16.24	8	61	0.95	0.03	9.42	4	33	1.00	0.00	10.37
*Klainodoxa gabonensis*	4	54	0.8	0.16	7.41	19	532	1.48	0.00	5.64					
*Maesobotria bateri*	13	602	0.83	0.00	5.17	3	26	0.00	0.00	7.32					
*Musanga cercopioides*	2	13	3.69	0.00	18.76	1	2	0.00	0.00	17.67	1	7	0.86	0.00	10.71
*Napoleona leonensis*	2	30	1.33	0.00	8.29	2	9	1.00	0.00	21.93					
*Parinari excelsa*	25	536	2.77	0.00	10.93	9	184	0.88	0.00	9.28	14	348	0.92	0.08	8.64
*Rothmannia whitjieldii*						13	181	0.97	0.04	10.43	6	138	0.66	0.25	9.33
*Sacoglottis gabonensis*	117	2057	2.98	0.00	17.36	59	781	0.96	0.00	9.73	15	338	0.70	0.00	17.65
*Uapaca esculenta*	11	360	1.36	0.00	10.31	2	37	0.91	0.09	11.72					
*Xylopia taiensis*	2	30	1.53	0.00	15.02	2	28	1.00	0.00	7.31					
**Leaves** **(young)**	235	2809	3.09	0.00	15.73	17	71	0.73	0.00	14.69	142	1136	0.56	0.00	17.24
*Craterispermum caudatum*	60	774	2.64	0.00	14.96	1	6	1.00	0.00	13.60	13	75	0.69	0.00	18.42
*Culcasia grandiflora*	16	143	4.84	0.00	23.69	5	18	0.86	0.00	16.53	16	87	0.77	0.00	26.53
*Maesobotria bateri*	48	575	2.76	0.00	14.69	4	15	0.79	0.00	18.33	36	311	0.67	0.00	15.77
*Pauridiantha sylvicola*	1	1	4.00	0.00	57.00						2	21	0.75	0.00	20.05
**Leaves** **(mature)**	350	4157	2.49	0.00	24.15	4	25	0.29	0.00	20.56	50	428	0.82	0.00	23.17
*Craterispermum caudatum*	74	848	2.57	0.00	23.86	1	5	0.00	0.00	18.00	5	28	0.60	0.00	38.40
*Maesobotria bateri*	30	440	2.11	0.00	22.12	2	10	0.38	0.00	24.06	22	230	0.78	0.00	18.31
**Invertebrates**	1164	9459	1.14	0.00	9.24	104	306	0.58	0.04	13.22	24	36	0.60	0.00	15.81
**All other**	14	376				10	43				5	28			
Fungi	8	160	1.17	0.00	11.44	1	1	0.00	0.00	28.00	3	20	0.25	0.00	17.20
*Diospyros manni* seeds						9	42	7.15	0.00	24.27	2	3	5.00	0.00	73.25
Flowers	1	159	1.06	0.00	1.45	1	2	1.00	0.00	11.00	1	17	0.06	0.00	4.53
*Trichosypha arborea* stems	5	80	1.51	0.00	7.39						1	6	1.00	0.00	12.33

**Table 4:** Results of resampling procedures carried out for species contrast in which the N was unequal between samples. Entries of NA (not applicable) for the *p*-value indicate that the food item was never observed being eaten by one of the contrasted taxa, or that having just a single focal observation from one or more taxa precluded a meaningful test.

	Incisions per Ingestive Action: Resampled Mean Pairwise Comparisons		Postcanine Chews per Ingestive Action: Resampled Mean Pairwise Comparisons	
	Species Comparison	*p*-Value	Species Comparison	*p*-Value
**Fruit**	*C. diana > C. campbelli*	0	*C. diana > C. campbelli*	0
	*C. diana > C. petaurista*	0	*C. diana > C. petaurista*	0
	*C. campbelli < C. petaurista*	0	*C. campbelli < C. petaurista*	0.04
*Cersalia afzeli* (ripe)	*C. diana > C. campbelli*	0	*C. diana > C. campbelli*	0.02
	*C. diana, C. petaurista*	NA	*C. diana, C. petaurista*	NA
	*C. campbelli, C. petaurista*	NA	*C. campbelli, C. petaurista*	NA
*Coelecarion oxycarpum* (ripe)	*C. diana > C. campbelli*	0	*C. diana = C. campbelli*	0.56
	*C. diana, C. petaurista*	NA	*C. diana, C. petaurista*	NA
	*C. campbelli, C. petaurista*	NA	*C. campbelli, C. petaurista*	NA
*Culcasia grandiflora* (ripe)	*C. diana, C. campbelli*	NA	*C. diana, C. campbelli*	NA
	*C. diana, C. petaurista*	NA	*C. diana, C. petaurista*	NA
	*C. campbelli < C. petaurista*	0	*C. campbelli < C. petaurista*	0
*Dialium aubrevillei* (ripe)	*C. diana > C. campbelli*	0	*C. diana > C. campbelli*	0
	*C. diana > C. petaurista*	0	*C. diana > C. petaurista*	0.01
	*C. campbelli = C. petaurista*	0.11	*C. campbelli < C. petaurista*	0.02
*Diospyros manni* (ripe)	*C. diana > C. campbelli*	0	*C. diana > C. campbelli*	0
	*C. diana > C. petaurista*	0	*C. diana > C. petaurista*	0
	*C. campbelli < C. petaurista*	0	*C. campbelli < C. petaurista*	0
*Diospyros sanza-minika* (ripe)	*C. diana > C. campbelli*	0	*C. diana > C. campbelli*	0
	*C. diana, C. petaurista*	NA	*C. diana, C. petaurista*	NA
	*C. campbelli, C. petaurista*	NA	*C. campbelli, C. petaurista*	NA
*Diospyros soubreana* (ripe)	*C. diana > C. campbelli*	0	*C. diana > C. campbelli*	0
	*C. diana > C. petaurista*	0	*C. diana > C. petaurista*	0
	*C. campbelli = C. petaurista*	0.49	*C. campbelli = C. petaurista*	0.14
*Klainodoxa gabonensis* (ripe)	*C. diana = C. campbelli*	0.12	*C. diana = C. campbelli*	0.12
	*C. diana, C. petaurista*	NA	*C. diana, C. petaurista*	NA
	*C. campbelli, C. petaurista*	NA	*C. campbelli, C. petaurista*	NA
*Maesobotria bateri* (ripe)	*C. diana > C. campbelli*	0	*C. diana > C. campbelli*	0
	*C. diana, C. petaurista*	NA	*C. diana, C. petaurista*	NA
	*C. campbelli, C. petaurista*	NA	*C. campbelli, C. petaurista*	NA
*Memecylon lateriflorum* (Unripe)	*C. diana, C. campbelli*	NA	*C. diana, C. campbelli*	NA
	*C. diana, C. petaurista*	NA	*C. diana, C. petaurista*	NA
	*C. campbelli < C. petaurista*	0	*C. campbelli < C. petaurista*	0
*Musanga cercopioides* (ripe)	*C. diana > C. campbelli*	0	*C. diana = C. campbelli*	0.15
	*C. diana > C. petaurista*	0	*C. diana = C. petaurista*	0.1
	*C. campbelli < C. petaurista*	0	*C. campbelli = C. petaurista*	0.48
*Napoleona leonensis* (ripe)	*C. diana > C. campbelli*	0	*C. diana > C. campbelli*	0
	*C. diana, C. petaurista*	NA	*C. diana, C. petaurista*	NA
	*C. campbelli, C. petaurista*	NA	*C. campbelli, C. petaurista*	NA
*Parinari excelsa* (ripe)	*C. diana > C. campbelli*	0	*C. diana = C. campbelli*	0.42
	*C. diana > C. petaurista*	0	*C. diana > C. petaurista*	0
	*C. campbelli = C. petaurista*	0.1	*C. campbelli > C. petaurista*	0
*Rothmannia whitjieldii* (ripe)	*C. diana, C. campbelli*	NA	*C. diana, C. campbelli*	NA
	*C. diana, C. petaurista*	NA	*C. diana, C. petaurista*	NA
	*C. campbelli = C. petaurista*	0.44	*C. cambelli < C. petaurista*	0
*Sacoglottis gabonensis* (ripe)	*C. diana > C. campbelli*	0	*C. diana > C. campbelli*	0
	*C. diana > C. petaurista*	0	*C. diana > C. petaurista*	0
	*C. cambelli < C. petaurista*	0	*C. campbelli = C. petaurista*	0.37
*Uapaca esculenta* (ripe)	*C. diana > C. campbelli*	0	*C. diana > C. campbelli*	0.01
	*C. diana > C. petaurista*	NA	*C. diana > C. petaurista*	NA
	*C. campbelli, C. petaurista*	NA	*C. campbelli, C. petaurista*	NA
*Xylopia taiensis* (ripe)	*C. diana = C. campbelli*	0.34	*C. diana = C. campbelli*	0.07
	*C. diana, C. petaurista*	NA	*C. diana > C. petaurista*	NA
	*C. campbelli, C. petaurista*	NA	*C. campbelli, C. petaurista*	NA
**Foliage**	*C. diana > C. campbelli*	0	*C. diana > C. campbelli*	0
	*C. diana > C. petaurista*	0	*C. diana > C. petaurista*	0
	*C. campbelli = C. petaurista*	0.23	*C. campbelli < C. petaurista*	0
*Craterispermum caudatum* (Mature)	*C. diana > C. campbelli*	0	*C. diana = C. campbelli*	0.06
	*C. diana > C. petaurista*	0	*C. diana = C. petaurista*	0.18
	*C. campbelli = C. petaurista*	0.44	*C. campbelli < C. petaurista*	0.04
*Culcasia grandiflora* (Young)	*C. diana > C. campbelli*	0	*C. diana > C. petaurista*	0.01
	*C. diana > C. petaurista*	0	*C. diana > C. petaurista*	0
	*C. campbelli = C. petaurista*	0.22	*C. campbelli < C. petaurista*	0.04
*Maesobotria bateri* (Young)	*C. diana > C. campbelli*	0	*C. diana > C. campbelli*	0.02
	*C. diana > C. petaurista*	0	*C. diana > C. petaurista*	0
	*C. campbelli = C. petaurista*	0.23	*C. campbelli < C. petaurista*	0.04
*Maesobotria bateri* (Mature)	*C. diana > C. campbelli*	0	*C. diana = C. campbelli*	0.40
	*C. diana > C. petaurista*	0	*C. diana > C. petaurista*	0
	*C. campbelli = C. petaurista*	0.15	*C. campbelli = C. petaurista*	0.07
*Trichosypha arborea* (petioles)	*C. diana, C. campbelli*	NA	*C. diana, C. campbelli*	NA
	*C. diana = C. petaurista*	0.59	*C. diana > C. petaurista*	0
	*C. campbelli, C. petaurista*	NA	*C. campbelli, C. petaurista*	NA
**Invertebrates**	*C. diana > C. campbelli*	0	*C. diana = C. campbelli*	0.22
	*C. diana > C. petaurista*	0	*C. diana = C. petaurista*	0.10
	*C. campbelli = C. petaurista*	0.38	*C. campbelli < C. petaurista*	0
**Fungi**	*C. diana, C. campbelli*	NA	*C. diana, C. campbelli*	NA
	*C. diana > C. petaurista*	0	*C. diana = C. petaurista*	0.17
	*C. campbelli, C. petaurista*	NA	*C. campbelli, C. petaurista*	NA
**Flowers**	*C. diana > C. campbelli*	0	*C. diana > C. campbelli*	0
	*C. diana > C. petaurista*	0	*C. diana = C. petaurista*	0.22
	*C. campbelli = C. petaurista*	0.41	*C. campbelli < C. petaurista*	0

## Data Availability

These data are available from the corresponding author upon request.

## Supplementary Materials

The following supporting information can be downloaded at https://www.mdpi.com/article/10.3390/biology11121850/s1, Table S1: Complete oral-processing data for all foods during our study period, Figure S1: Incisions per ingestive action of individual Diana monkeys and Campbell’s monkeys while eating the same foods, and Figure S2: Post-canine chews per ingestive action of individual Diana monkeys and Campbell’s monkeys while eating the same foods.
