# Supplementary material for: Oral Processing of Three Guenon Species in Taï National Park, Côte d’Ivoire
Source: Biology (Basel). 2022 Dec 19;11(12):1850. doi: 10.3390/biology11121850 (PMC9775981; doi:10.3390/biology11121850)
Supplement: Supplementary file 1 [file biology-11-01850-s001.zip › biology-2030225-supplementary.pdf]

Supplementary Materials

Species	*C. campbelli*					*C. diana*					*C. petaurista*				
	Focals (N)	Action(N)	I[1]/A	C[2]/A	PC[3]/A	Focals (N)	Action(N)	I/A	C/A	PC/A	Focals (N)	Action(N)	I/A	C/A	PC/A
**Fruit**	223	2574	0.86	0.01	10.15	582	11657	2.07	0.00	19.48	73	1156	0.88	0.05	9.91
Annikia polycarpa						1	3	0.00	0.00	18.00					
Aninguera robusta						2	28	1.55	0.00	6.82					
Ans. mam	1	6	1.00	0.00	18.83										
Calpocalyps brevibactus						2	14	2.50	0.00	16.53					
Cersalia afzeli	4	13	0.88	0.00	10.43	1	8	1.63	0.00	26.38					
Cissus sp.	1	14	0.00	0.00	7.29										
Coelycarion oxycarpum	1	12	1.00	0.00	5.50	1	10	1.10	0.00	4.30					
Culcasia grandiflora	1	21	1.00	0.00	2.80						2	11	1.00	0.00	25.13
Dacryodes klaineana						17	428	1.25	0.00	3.78					
Dialium aubrevillei	15	156	0.91	0.00	6.96	27	789	1.30	0.00	8.90	7	92	0.85	0.00	7.21
Dialium dinklagei	1	3	1.00	0.00	17.00										
Diospyros manni	25	241	0.95	0.02	7.05	148	2547	1.92	0.00	37.74	7	78	0.85	0.15	11.32
Diospyros sanza-minika	3	33	1.00	0.00	5.75	22	305	1.90	0.00	21.14					
Diospyros soubreana	8	61	0.95	0.03	9.42	61	936	4.58	0.00	16.24	4	33	1.00	0.00	10.37
Heritiera utilis	3	19	0.93	0.00	5.52										
Klainodoxa gabonensis	4	54	0.8	0.16	7.41	19	532	1.48	0.00	5.64					
Landolphia welwitschii						1	13	1.23	0.00	11.54					
Maesobotria bateri	3	26	0.00	0.00	7.32	13	602	0.83	0.00	5.17					
Memecylon lateriflorum	13	112	0.85	0.00	25.54										
Musanga cercopioides	1	2	0.00	0.00	17.67	2	13	3.69	0.00	18.76	1	7	0.86	0.00	10.71
Napoleona leonensis	2	9	1.00	0.00	21.93	2	30	1.33	0.00	8.29					
Naulea pobeguini	1	5	1.00	0.00	29.20										
Octonema borealis						2	15	1.33	0.00	3.04					
Parinari aubrevillei	1	16	1.41	0.00	3.71										
Parinari excelsa	9	184	0.88	0.00	9.28	25	536	2.77	0.00	10.93	14	348	0.92	0.08	8.64
Parkia bicolor						5	76	2.29	0.00	8.12					
Pauridiantha sylvicola						1	7	1.00	0.00	1.71					

Pentadesma butacyrea						1	19	1.26	0.00	3.32					
Pycnanthus angolensis						3	35	1.52	0.00	11.75					
Rothmannia whitjieldii	13	181	0.97	0.04	10.43						6	138	0.66	0.25	9.33
Sacoglottis gabonensis	59	781	0.96	0.00	9.73	117	2057	2.98	0.00	17.36	15	338	0.70	0.00	17.65
Salacia leteritia						3	63	1.42	0.00	12.70					
Scytopetalum tieghemii						74	1421	0.18	0.00	13.01					
Spondianthus preussi											1	12	0.58	0.00	0.67
Spyropetalum sp.						4	187	1.05	0.00	3.78					
Sterculia tragacantha	2	13	1.00	0.00	12.55										
Tetracera potatoria											2	40	0.56	0.00	11.94
Trichosypha arborea						3	107	1.12	0.00	5.13					
Trichosypha beguei	8	89	0.80	0.00	9.62										
Uapaca esculenta	2	37	0.91	0.09	11.72	11	360	1.36	0.00	10.31					
Uapaca guinensis	14	188	0.93	0.00	13.80										
Xylopia parviflora	1	6	1.00	0.00	6.50										
Xylopia taiensis	2	28	1.00	0.00	7.31	2	30	1.53	0.00	15.02					
**Unripe fruit**															
Culcasia grandiflora	1	9	1.00	0.00	4.56										
Diospyros manni	1	8	1.00	0.00	5.88										
Diospyros soubreana	4	63	0.75	0.00	8.31										
Heritiera utilis	1	5	1.00	0.00	16.00										
Hypselodelphys violacea											1	10	0.90	0.00	13.50
Maesobotria bateri	11	129	0.45	0.00	7.42										
Memecylon lateriflorum											3	43	1.00	0.00	16.52
Napoleona leonensis	4	29	0.75	0.00	6.21										
Sterculia tragacantha	3	21	1.00	0.00	11.50										
Warneckea membranifoli											1	6	1.00	0.00	16.50
**Young leaves**	17	71	0.73	0.00	14.69	235	2809	3.09	0.00	15.73	142	1136	0.56	0.00	17.24
Calpocalyx brevibactus											1	6	0.00	0.00	13.67
Cepheoli yapoinsis						2	28	2.01	0.00	16.63					
Cersalia afzeli						23	174	4.19	0.00	21.76					
Combretum aphanoptealum											10	58	0.33	0.00	23.39

Craterispermum caudatum	1	6	1.00	0.00	13.60	60	774	2.64	0.00	14.96	13	75	0.69	0.00	18.42
Culcasia grandiflora	5	18	0.86	0.00	16.53	16	143	4.84	0.00	23.69	16	87	0.77	0.00	26.53
Dalbergia cyclocarpa											8	63	0.36	0.00	15.73
Dialium aubrevillei	2	7	0.54	0.00	11.63										
Griffonia simplicifolia											2	15	0.72	0.00	12.97
Maesobotria bateri	4	15	0.79	0.00	18.33	48	575	2.76	0.00	14.69	36	311	0.67	0.00	15.77
Memecylon lateriflorum											4	35	0.51	0.00	16.49
Pauridiantha sylvicola						1	1	4.00	0.00	57.00	2	21	0.75	0.00	20.05
Rhigiocarya racemfera											1	7	0.29	0.00	11.00
Strychnos aculeata											1	12	1.00	0.00	13.25
Vitex micrantha											4	53	0.61	0.00	20.68
Mature leaves	4	25	0.29	0.00	20.56	350	4157	2.49	0.00	24.15	50	428	0.82	0.00	23.17
Cepheoli yapoinsis						1	24	1.08	0.00	10.46					
Craterispermum caudatum	1	5	0.00	0.00	18.00	74	848	2.57	0.00	23.86	5	28	0.60	0.00	38.40
Culcasia grandiflora											2	14	1.00	0.00	24.98
Dalbergia cyclocarpa											1	4	0.75	0.00	26.75
Maesobotria bateri	2	10	0.38	0.00	24.06	30	440	2.11	0.00	22.12	22	230	0.78	0.00	18.31
Napoleona leonensis						1	10	1.00	0.00	21.20					
Pauridiantha sylvicola											3	29	0.97	0.00	27.43
Polyathia olivieri											8	48	0.98	0.00	28.16
Vitex grandifolia											1	11	0.55	0.00	21.43
Vitex micrantha											1	4	0.75	0.00	12.75
Invertebrates	104	306	0.58	0.04	13.22	1164	9459	1.14	0.00	9.24	24	36	0.60	0.00	15.81
Other material															
Craterispermum caudatum flower						1	159	1.06	0.00	1.45	1	17	0.06	0.00	4.53
Polyathia olivieri flower											2	26	0.00	0.00	19.02
Popovia flower	1	2	1.00	0.00	11.00										
Rothmannia whitjieldii flower											4	20	1.00	0.00	19.80
Frog eggs						8	125	1.86	0.00	3.36					
Mushroom	1	1	0.00	0.00	28.00	8	160	1.17	0.00	11.44	3	20	0.35	0	19.53
Sap						3	15	2.04	0.00	17.60					
Diospyros manni seed	9	42	7.15	0.00	24.27						2	3	5.00	0.00	73.25

Diospyros soubreana seed	2	16	0.50	0.00	6.76										
Strephonyma pseudocola seed	3	11	0.47	0.00	7.61						1	5	0.00	0.00	22.00
Dalbergia cyclocarpa stem	1	3	0.67	0.00	24.00										
Diospyros canaliculata stem											4	60	0.98	0.00	7.12
Heritiera utilis stem	1	11	1.00	0.00	16.82										
Palisota hirsuta stem	5	82	0.99	0.00	10.82										
Polyathia olivieri stem											2	35	0.74	0.26	8.92
Sacoglottis gabonensis stem											1	2	1.00	0.00	3.50
Trichosypha arborea stem						5	80	1.51	0.00	7.39	1	6	1.00	0.00	12.33
Termite mound	1	1	5.00	0.00	37.00										

Supplementary Table S1. We report the number of focals by food type and by species, the number of ingestive actions, and mean incisor use, canine use, and post-canine chews per ingestive action for all identified food items eaten by each guenon species. Total values and means for broad food categories include unidentified plant species (e.g., unknown fruit, young leaves of unknown liana) as well as identified food items.

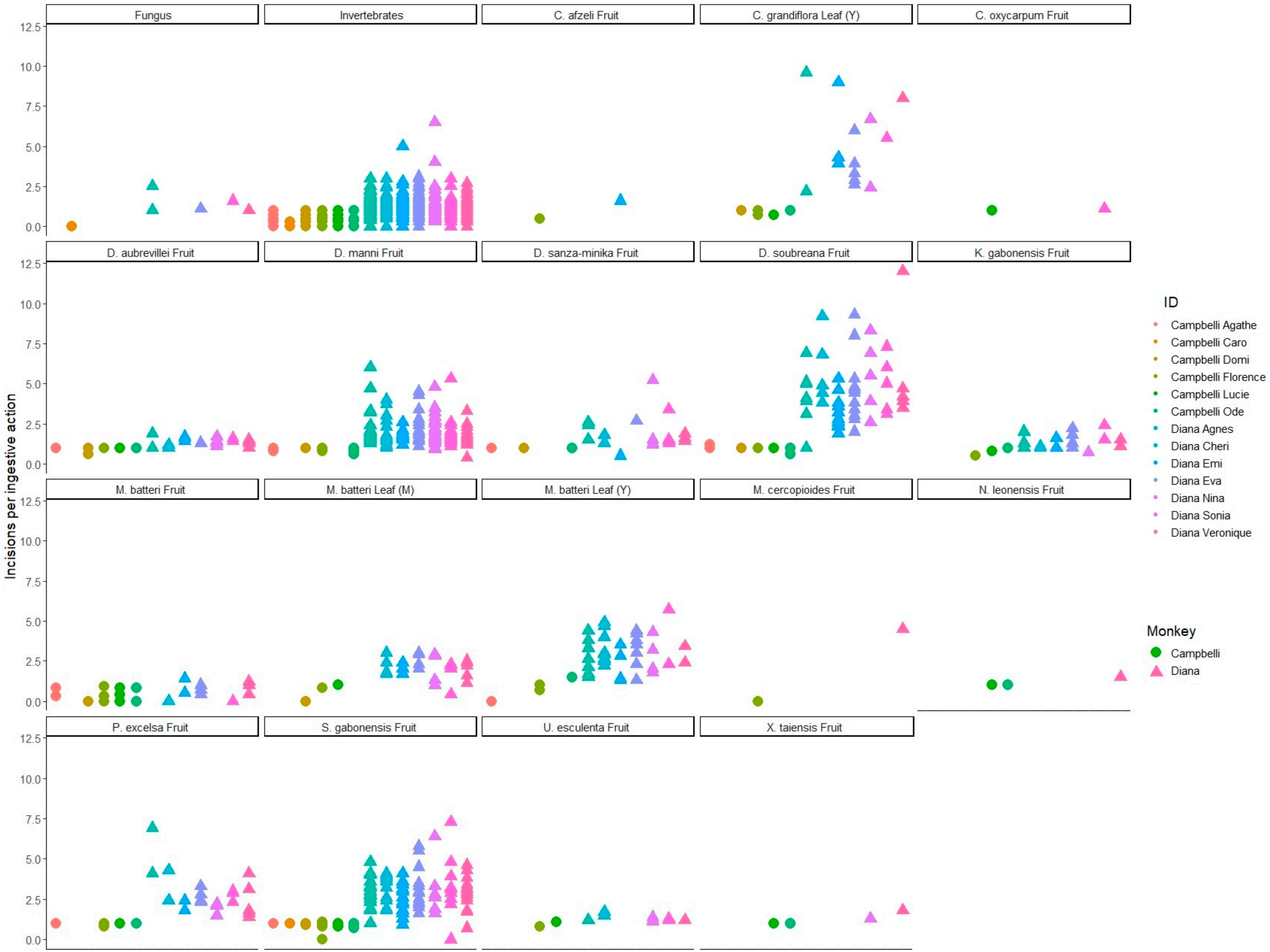

Supplemental Figure S1. Incisions per ingestive action during focal follows of individually identified Campbell's and Diana monkeys feeding on shared food items.

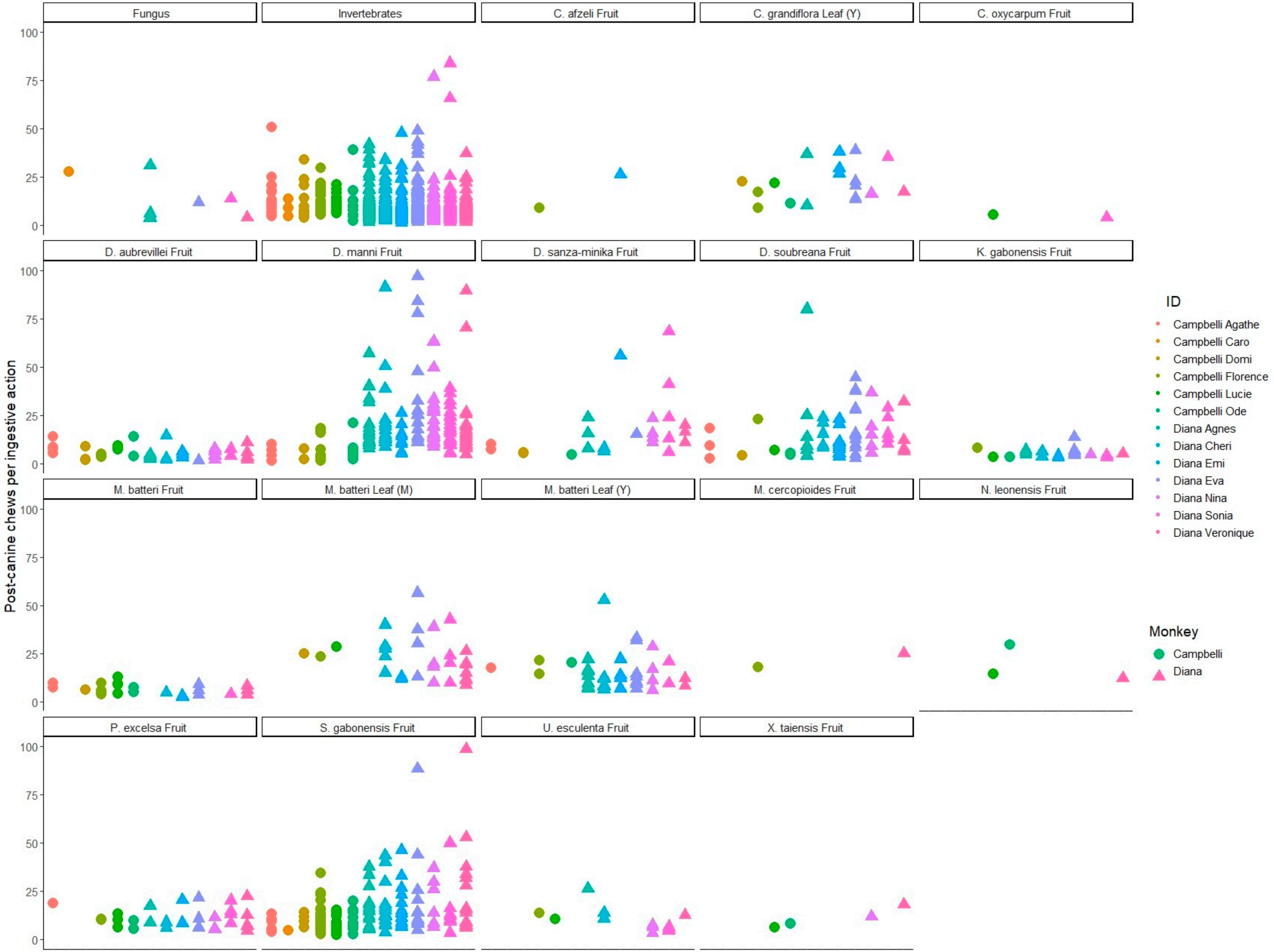

Supplemental Figure S2. Post-canine chews per ingestive action during focal follows of individually identified Campbell's and Diana monkeys feeding on shared food items.
